# Motor-cognitive functions required for driving in post-stroke individuals identified via machine-learning analysis

**DOI:** 10.1186/s12984-023-01263-z

**Published:** 2023-10-18

**Authors:** Genta Tabuchi, Akira Furui, Seiji Hama, Akiko Yanagawa, Koji Shimonaga, Ziqiang Xu, Zu Soh, Harutoyo Hirano, Toshio Tsuji

**Affiliations:** 1 Graduate School of Engineering, Hiroshima University, 1-4-1 Kagamiyama, Higashi-Hiroshima, Hiroshima 739-8527 Japan; 2 Graduate School of Advanced Science and Engineering, Hiroshima University, 1-4-1 Kagamiyama, Higashi-Hiroshima, Hiroshima 739-8527 Japan; 3 Graduate School of Biomedical and Health Sciences, Hiroshima University, 1-2-3 Kasumi, Minami-ku, Hiroshima, Hiroshima 734-8551 Japan; 4 Department of Rehabilitation, Hibino Hospital, 7-9-2 Tomo-Higashi, Asaminami-ku, Hiroshima, Hiroshima 731-3164 Japan; 5 Department of Neurosurgery and Interventional Neuroradiology, Hiroshima City North Medical Center Asa Citizens Hospital, 1-2-1 Kameyamaminami, Asakita-ku, Hiroshima, Hiroshima 731-0293 Japan; 6 Department of Medical Equipment Engineering, Clinical Collaboration Unit, School of Medical Sciences, Fujita Health University, 1-98 Dengakugakubo, Kutsukake-cho, Toyoake, Aichi 470-1192 Japan

**Keywords:** Driving aptitude, Machine-learning method, Motor-cognitive functions, Post-stroke individuals

## Abstract

**Background:**

People who were previously hospitalised with stroke may have difficulty operating a motor vehicle, and their driving aptitude needs to be evaluated to prevent traffic accidents in today’s car-based society. Although the association between motor-cognitive functions and driving aptitude has been extensively studied, motor-cognitive functions required for driving have not been elucidated.

**Methods:**

In this paper, we propose a machine-learning algorithm that introduces sparse regularization to automatically select driving aptitude-related indices from 65 input indices obtained from 10 tests of motor-cognitive function conducted on 55 participants with stroke. Indices related to driving aptitude and their required tests can be identified based on the output probability of the presence or absence of driving aptitude to provide evidence for identifying subjects who must undergo the on-road driving test. We also analyzed the importance of the indices of motor-cognitive function tests in evaluating driving aptitude to further clarify the relationship between motor-cognitive function and driving aptitude.

**Results:**

The experimental results showed that the proposed method achieved predictive evaluation of the presence or absence of driving aptitude with high accuracy (area under curve 0.946) and identified a group of indices of motor-cognitive function tests that are strongly related to driving aptitude.

**Conclusions:**

The proposed method is able to effectively and accurately unravel driving-related motor-cognitive functions from a panoply of test results, allowing for autonomous evaluation of driving aptitude in post-stroke individuals. This has the potential to reduce the number of screening tests required and the corresponding clinical workload, further improving personal and public safety and the quality of life of individuals with stroke.

**Supplementary Information:**

The online version contains supplementary material available at 10.1186/s12984-023-01263-z.

## Introduction

Approximately 1.1 million individuals in Japan have suffered from stroke [1], which manifests via various symptoms, including paresis, aphasia, and cognitive function, depending on the location of the lesion. Among these symptoms, more than two-thirds of post-stroke individuals are thought to suffer from some degree of cognitive disturbances [2], resulting in interfere with activities of daily living (ADLs) and the need for additional care [3]. Even if post-stroke individuals are able to engage in ADLs, their social life can be severely impacted. Driving a car is an effective way for people with impaired walking ability to participate in social activities [4]; however, driving is a complex task that requires many cognitive and executive functions to regulate physical behaviors [5, 6]. On the other hand, the main causes of traffic accidents among drivers include incorrect perception, poor judgment, and the inability to respond to emergencies in a timely manner. These risk factors can be exacerbated by stroke or dementia with age [7], as evidenced by the increasing number of traffic accidents compared to the control group depending on the type of stroke [2]. In addition, traffic accidents caused by the elderly and people with dementia has increased in recent years. Therefore, for many individuals with cognitive impairment due to these disorders, evaluations of driving aptitude are required for today’s car-based society.

On-road driving tests using a real car are the gold standard for evaluating driving aptitude and previous studies showed no increase in the number of car accidents among post-stroke drivers who passed the test compared to the control group [8]. Whereas it is difficult to perform on-road test to all participants with stroke, there is a need to screen for the necessity of road testing. Therefore, in medical institutions, evaluation with a driving simulator and physical and cognitive function tests are also used [9]. A driving aptitude evaluation method using a simulator to reproduce on-road driving test situations has been developed. The simulator evaluation can provide an evaluation of situations that cannot be evaluated using on-road testing, such as bad weather, collision avoidance, and abrupt changes in the road [2]. However, there are various types of simulators with different standards, making it difficult to obtain better and constant results than on-road testing [2].

Physical and cognitive function tests have been performed in medical institutions to perform rehabilitation for individuals with stroke. Several studies have investigated the association between physical and cognitive function tests and driving aptitude [9–11]. Reger et al. reported relationships among the results of cognitive function tests, including attention, visuospatial cognition, memory, and executive function, and the results of on-road testing and the evaluation results from a driving simulator [10]. Yamada et al. performed two cognitive function tests, the mini-mental state examination (MMSE) and the trail-making test (TMT), on participants with stroke and reported relationships between the results of tests and driving aptitude [11]. A number of physical and/or cognitive function tests have shown the significant relation with driving aptitude, while too few studies have supported their reliability and validity in predicting driving aptitude. It was because that there are too many cognitive tests for measuring various cognitive dysfunction after stroke, and very few reports examined by the single method and verified the predictive ability of operation aptitude. As a result, there is no consensus regarding a valid and reliable test to determine the driving aptitude for individuals with stroke. Therefore, there is a need at this stage to conduct on-road driving tests to judge driving aptitude in subjects with hospital screening subtractions [2].

This paper investigates the association between driving aptitude and physical impairment, and cognitive dysfunction in participants with stroke. In this study, 10 tests of physical and cognitive function were administered to participants with stroke, taking up to 5 h per participant and lasting a week or more to complete. Therefore, it is necessary to use it as screening after reducing the number of essential tasks out of a physical-cognitive function test group. Here, we proposed a machine learning algorithm that can automatically select input indices related to driving aptitude by introducing sparse regularization, aiming to develop an effective method to identify subjects who must undergo the on-road driving test through prior hospital-based tests. In addition, we also analyzed the relationship between the indices obtained from physical and cognitive tests and driving aptitude using the proposed algorithm, thus promising to provide a theoretical basis for further valid evaluation.

**Table 1:** Clinical characteristic of the participants

	Participants ($n=55$)	Drivable group ($n=41$)	Undrivable group ($n=14$)	*p*-value
Age (years)	61.4 ± 11.3	61.2 ± 11.4	61.7 ± 11.2	0.681
Sex (male), *n* (%)	48 (87.3%)	35 (85.4%)	13 (92.6%)	0.664
Period from onset				
to driving school (days)	182.3 ± 165.0	155.9 ± 121.7	259.6 ± 242.6	0.143
Disease				
Infarction, *n* (%)	36 (65.5%)	28 (68.3%)	8 (57.1%)	0.522
Hemorrhage, *n* (%)	20 (36.3%)	14 (34.1%)	6 (42.9%)	0.749
Stroke severity				
Motor FIM on admission	59.2 ± 20.7	62.4 ± 20.6	48.2 ± 17.6	**0.0095**
Cognitive FIM on admission	26.6 ± 6.9	27.2 ± 7.3	24.7 ± 4.7	**0.0329**
Motor FIM on discharge	81.3 ± 9.6	82.4 ± 8.9	77.6 ± 11.1	**0.0386**
Cognitive FIM on discharge	31.0 ± 4.4	31.6 ± 4.5	29.1 ± 3.8	**0.015**
Motor FIM improvement rate	22.0 ± 16.8	20.0 ± 17.2	29.1 ± 13.6	**0.0291**
Cognitive FIM improvement	4.4 ± 5.8	4.4 ± 6.0	4.4 ± 5.2	0.7703
Physical disability				
BRS total score on admission	12.9 ± 5.8	13.8 ± 5.4	10.4 ± 6.3	0.1382
BRS total score on discharge	15.6 ± 3.8	16.0 ± 3.6	14.5 ± 4.4	0.1661
Dominant side Left, *n* (%)	5 (9.1%)	5 (12.2%)	0 (0%)	0.314
Dominant side Right, *n* (%)	52 (94.6%)	38 (92.7%)	14 (100%)	0.562
Affected side Left, *n* (%)	28 (50.9%)	17 (41.5%)	11 (78.6%)	**0.0286**
Affected side Right, *n* (%)	27 (49.1%)	24 (58.5%)	3 (21.4%)	**0.0286**
Ataxia, *n* (%)	3 (5.5%)	3 (7.3%)	0 (0%)	0.562
Dysarthria, *n* (%)	2 (3.6%)	2 (4.9%)	0 (0%)	1
Aphasia, *n* (%)	4 (7.3%)	2 (4.9%)	2 (14.3%)	0.265
Hospitalization period (days)	70.8 ± 45.3	64.2 ± 37.9	90.1 ± 59.6	0.186
Psychological assessment				
HADS-Depression	4.9 ± 3.5	4.3 ± 3.5	6.6 ± 3.3	**0.0298**
HADS-Anxiety	5.3 ± 3.3	5.3 ± 3.5	5.3 ± 3.1	0.9058
Apathy score	11.7 ± 5.8	11.4 ± 5.5	12.5 ± 6.9	0.6327
JPSS	20.2 ± 6.0	19.7 ± 6.1	21.6 ± 5.5	0.3839
Cognitive function test				
MMSE: Orientation-time	4.6 ± 0.7	4.9 ± 0.4	4.0 ± 1.0	**0.0004**
MMSE: Orientation-location	4.8 ± 0.5	4.8 ± 0.5	4.7 ± 0.5	0.4647
MMSE: Attention and calculation	3.8 ± 1.7	3.9 ± 1.7	3.6 ± 1.6	0.4967
MMSE: Recall	2.6 ± 0.6	2.6 ± 0.6	2.7 ± 0.6	0.4985
MMSE: Naming	2.0 ± 0.3	2.0 ± 0.3	2.0 ± 0	0.3233
MMSE: Repetition, *n* (%)	52 (94.6%)	38 (92.7%)	14 (100%)	0.562
MMSE: 3-stage command	3.0 ± 0.2	3.0 ± 0.2	3.0 ± 0.0	0.1598
MMSE: Writing, *n* (%)	50 (90.9%)	38 (92.7%)	12 (85.7%)	0.592
MMSE: Copying, *n* (%)	45 (81.8%)	34 (82.9%)	11 (78.6%)	0.703
MMSE total score	27.5 ± 2.7	27.7 ± 2.6	26.7 ± 2.9	0.2129
TMT part A time	59.7 ± 40.0	51.8 ± 22.2	82.9 ± 65.9	**0.0051**
Digit span forward	5.6 ± 1.0	5.6 ± 0.1	5.4 ± 1.0	0.8395
Digit span backward	4.0 ± 1.3	4.0 ± 1.5	3.7 ± 0.8	0.4963
Tapping span forward	5.6 ± 1.4	5.9 ± 1.3	4.8 ± 1.7	**0.0166**
Tapping span backward	5.1 ± 1.5	5.4 ± 1.4	4.4 ± 1.5	**0.0250**
Visual cancellation Kana time	145.5 ± 38.0	146.7 ± 36.7	141.8 ± 42.7	0.7739
Visual cancellation $\triangle$ time	70.2 ± 24.3	67.8 ± 20.7	77.2 ± 32.6	0.391
Visual cancellation star time	78.0 ± 20.2	77.1 ± 21.5	80.8 ± 16.0	0.4058
Visual cancellation figure time	120.2 ± 35.0	120.3 ± 34.0	119.9 ± 39.1	0.9567
Visual cancellation Kana accuracy	93.4 ± 11.7	95.9 ± 5.3	85.8 ± 20.0	**0.0094**
Visual cancellation Kana hit rate	99.5 ± 1.8	99.7 ± 1.5	99.2 ± 2.5	0.4411
Visual cancellation $\triangle$ accuracy	97.2 ± 7.9	98.5 ± 2.0	93.3 ± 15.0	0.0770
Visual cancellation $\triangle$ hit rate	99.0 ± 3.6	99.6 ± 1.2	97.2 ± 6.8	0.5435
Visual cancellation star accuracy	97.7 ± 9.3	99.3 ± 1.7	93.1 ± 17.9	**0.0218**
Visual cancellation star hit rate	99.4 ± 2.6	99.6 ± 1.1	98.7 ± 4.9	0.5124
Visual cancellation figure accuracy	97.3 ± 10.7	99.0 ± 1.4	92.3 ± 20.9	**0.0108**
Visual cancellation figure hit rate	99.9 ± 0.6	99.9 ± 0.2	99.7 ± 1.1	0.7517
SDMT number of wrong answers	1.2 ± 2.0	1.2 ± 2.1	1.1 ± 1.9	0.6637
SDMT acheivement rate	32.9 ± 10.2	34.7 ± 9.3	27.6 ± 11.3	**0.0334**
Memory updating 3 span accuracy	60.2 ± 21.3	62.5 ± 22.1	53.6 ± 18.0	0.1144
PASAT 2 seconds accuracy	45.6 ± 17.0	47.5 ± 17.5	40.3 ± 14.7	0.1432
Position stroop accuracy	96.5 ± 8.6	97.7 ± 3.7	92.8 ± 15.6	0.977
Position stroop time	115.4 ± 40.4	110.2 ± 28.0	130.6 ± 63.6	0.1832
CPTAX accuracy	93.7 ± 9.1	95.1 ± 7.8	89.7 ± 11.7	0.0572
CPTAX hit rate	89.2 ± 14.2	91.1 ± 12.1	83.6 ± 18.5	0.1203
CPTAX average reaction time	584.7 ± 88.5	578.9 ± 93.1	601.6 ± 74.1	0.3051
CPTAX coefficient of validation	17.4 ± 4.6	17.3 ± 4.3	17.9 ± 5.5	0.661
CPTSRT accuracy	95.8 ± 9.4	98.3 ± 1.9	88.5 ± 16.6	**0.0168**
CPTSRT hit rate	97.1 ± 8.4	98.9 ± 1.4	91.8 ± 15.7	**0.0085**
CPTSRT average reaction time	386.1 ± 96.3	362.0 ± 70.2	456.5 ± 127.4	**0.0499**
CPTSRT coefficient of validation	21.5 ± 6.7	21.7 ± 6.8	20.7 ± 6.7	0.7512
CPTX accuracy	95.8 ± 8.7	98.3 ± 3.9	88.6 ± 13.9	**0.0001**
CPTX hit rate	94.0 ± 11.6	96.5 ± 5.9	86.4 ± 19.3	**0.0042**
CPTX average reaction time	564.8 ± 89.1	549.0 ± 78.9	610.8 ± 103.6	0.0572
CPTX coefficient of validation	14.9 ± 4.5	14.6 ± 4.7	15.6 ± 3.9	0.266
Line crossing test	36.0 ± 0.3	36.0 ± 0.2	35.9 ± 0.5	0.5335
Letter cancellation test	37.4 ± 3.2	38.0 ± 2.4	35.6 ± 4.4	**0.0159**
Star cancellation test	52.8 ± 4.0	53.6 ± 0.7	50.3 ± 7.4	**0.0434**
Copying test	3.4 ± 1.1	3.7 ± 0.6	2.4 ± 1.5	**0.0058**
Line bisection test	8.6 ± 1.4	8.9 ± 0.5	7.9 ± 2.5	0.1215
Drawing test	2.8 ± 0.7	2.9 ± 0.6	2.5 ± 0.9	0.0736
BIT conventional subtest	140.8 ± 8.1	142.9 ± 3.2	134.6 ± 13.7	**0.0054**
BIT behavioral subtest	77.9 ± 7.8	79.1 ± 2.5	74.4 ± 14.7	0.1087
RBMT profile	19.5 ± 3.7	20.2 ± 3.5	17.4 ± 3.3	**0.0003**

All results are presented as Mean ± SD or number (%). Differences between the two groups with and without driving aptitude were examined using the Fisher’s exact test (categorical values) and Brunner-Munzel test (continuous values), and significant *p*-values ($<0.05$) are in bold

FIM: Functional independence measure; BRS: Brunnstorm stages of motor recovery; HADS: Hospital anxiety and depression scale; JPSS: Japanese perceived stress scale; MMSE: mini-mental state examination; TMT: Trail making test; SDMT: Symbol digit modalities test; PASAT: Paced auditory serial addition test; CPTAX: Continuous performance test AX version; CPTSRT: Continuous performance test simple version; CPTX: Continuous performance test X version; BIT: Behavioural inattention test; RBMT: Revermead behavioural memory test

## Materials and methods

### Participant data

Fifty-five participants with stroke (age: 61.3 ± 11.3 years) of Hibino Hospital participated in this study. Of these, 41 participants (age: 61.7 ± 11.2 years) were evaluated as a group with driving aptitude, and 14 participants (age: 61.2 ± 11.4 years) were evaluated as a group without driving aptitude. Table 1 shows the baseline data for participants in the groups with and without driving aptitude. The participants were asked to take the following 10 types of tests: the functional independence measure (FIM) [12], a type of functional independence assessment method; MMSE [13, 14], TMT [15, 16], which are cognitive function tests; Rivermead behavioral memory test (RBMT) [17], which is a type of memory test; CAT [18], which indicates attention function; the Japanese perceived stress scale (JPSS) [19, 20], which is a type of self-perceived stress test; the Brunnstrom stages of motor recovery (BRS) [21], which indicates the degree of paralysis; dominant hand side before stroke and affected side after stroke were also included in this analysis; the BIT [22], which indicates the degree of unilateral spatial neglect; the hospital anxiety and depression scale (HADS) [23, 24], which determines the presence or absence of depression and anxiety; the apathy score (AS) [25, 26], which determines the presence or absence of apathy.

The participants performed an on-road driving test and simulator test at the Numata driving school to evaluate their driving aptitude as described previously [27]. Driving aptitude was evaluated by driving school instructors based on the results of these tests. Based on the driving simulator results and the on-road testing, the participants were classified into two groups: a group with driving aptitude that the driving school instructor judged to be able to drive, and a group assessed to be unable to drive or require further training. The study was approved by the Ethics Review Committee of the Hiroshima University Epidemiological Research (E-1554-2, E-466-3) and was performed per relevant guidelines and regulations. Written informed consent was obtained from all participants.

### Proposed neural network model

The log-linearized Gaussian mixture network (LLGMN) [28], a neural network based on a discriminative Gaussian mixture model, can estimate the posterior probability of the target class that the input data belong to by estimating the statistical distribution of the sample data. So far, several studies have demonstrated its effectiveness in recognizing biological information [29, 30], including an application to analyze the relationship between driving aptitude and physical and cognitive functions [31]. Moreover, since only the number of mixtures is a hyperparameter, the tuning cost is lower than that of general neural networks such as the multilayer perceptron [32]. However, simply evaluating driving aptitude using the LLGMN does not reveal the relationship between each participant’s clinical index and driving aptitude, rendering it challenging to realize the original intent of establishing an effective method for identifying subjects who must undergo an on-road driving test through hospital-based physical and cognitive function screening tests. Therefore, this study proposes an LLGMN-based sparse neural network that can automatically select indices related to driving aptitude by adding a dimensionality reduction layer with sparse weights before the input layer of the LLGMN.

**Fig. 1:**
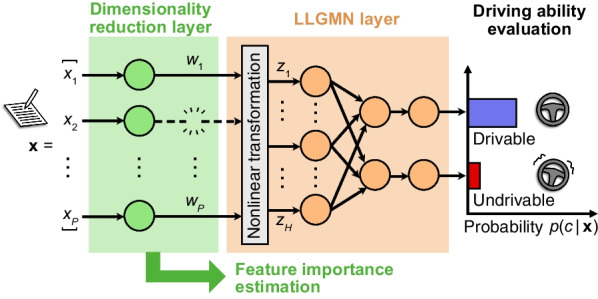
Overview of the proposed neural network model for evaluating driving aptitude. The proposed network is composed of two parts: a dimensionality reduction layer with $L_1$ regularized weights and a log-linearized Gaussian mixture network (LLGMN) [28]. This network calculates the posterior probability $p(c|\textbf{x})$ of the presence or absence of driving aptitude (i.e., drivable or undrivable) using the indices $\textbf{x}$ obtained from the physical and cognitive function tests as input. The weight parameters of the dimensionality reduction layer are denoted by $\textbf{w} = \{w_i\}_{i=1}^P$

The overview of the proposed network is shown in Fig. 1. The network’s input is a *P* dimensional index value $\textbf{x} \in \mathbb {R}^P$ obtained from 10 physical and cognitive tests. The output is the posterior probability $p(c|\textbf{x})$ of class $c \in \{1, 2\}$ representing the presence or absence of driving aptitude. The following equation can express the relationship between the input and output in the dimensionality reduction layer:

1 $$\begin{aligned} y_i=w_i x_i \ \ \ (i=1,2,...,P), \end{aligned}$$

where $y_i$ is the dimensionality reduction layer’s output, and $w_i$ is the dimensionality reduction layer’s weights. In the proposed network, the corresponding input dimension is reduced by sparsifying the dimensionality reduction layer’s weights.

Given a set of training data $\textbf{x}_n$ and corresponding target values $t_{nc}$ indicating the presence or absence of driving aptitudes ($n = 1, \ldots , N$; *N* is the number of participants for learning), let us consider the training of the proposed network. The target $t_{nc}$ is a vector of one-of-*C* form, indicating the presence or absence of driving aptitude, where $t_{nc} = 1$ if $\textbf{x}_n$ belongs to class *c*, $t_{nc} = 0$ otherwise. The solution of the proposed network can be obtained at the same time as learning by applying $L_1$ regularization to the weights $w_i$ of the dimensionality reduction layer. Here, the energy function *E* of the proposed network is defined as follows:

2 $$\begin{aligned} E = -\sum ^{N}_{n=1}\sum ^{2}_{c=1} t_{nc} \ln p(c|\textbf{x}_n)+\lambda \sum _{i=1}^{P} |w_i|, \end{aligned}$$

where the first term represents the cross-entropy error and the second term is the $L_1$ regularization term, which penalizes the error according to the magnitude of the absolute value of the dimensionality reduction layer’s weights ($w_i$). The parameter $\lambda$ is a regularization coefficient that determines the strength of $L_1$ regularization.

The proposed network minimizes the energy function *E* based on the error backpropagation method. Hence, the parameters of the entire network can be learned in an end-to-end fashion to reduce the error within the range where $w_i$ does not become large. Some $w_i$ will be completely zero at the end of training by applying $L_1$ regularization to the dimensionality reduction layer’s weights. Thus, input indices corresponding to $w_i$ that become zero can be excluded from the model, and input indices related to driving aptitude can be selected automatically. As a result, redundant input dimensions may be minimized, further identifying crucial indices that are highly relevant to the evaluation of driving aptitude and reducing test time in future applications.

### Importance of indices

It is important to identify the key features of a model in relational analysis using machine learning. $L_1$ regularization is applied to $w_i$ in the dimensionality reduction layer in the proposed network to perform parameter reduction estimation and dimensionality reduction by actively reducing the $w_i$ with a low contribution to zero error reduction. The $w_i$ remaining after learning represents the contribution of each index to the discrimination result; hence, the importance of each index may be obtained by examining the magnitude of $w_i$.

We also examined the importance of indices using the permutation importance [33], which is one way to evaluate the importance of machine learning inputs, and compared it with the learned $w_i$ in the proposed network. The permutation importance used the following algorithm to evaluate the importance of indices.

1. After splitting the whole dataset into training and validation datasets based on the cross-validation procedure, the model is trained using the training dataset.
2. The validation data, $\textbf{X}^\text{val} = \{\textbf{x}^\text{val}_n\}$ ($n = 1, 2, \ldots ,N^\text{val}$; $N^\text{val}$ is the number of participants in the validation data), are input to the trained model to calculate the cross-entropy error *e* which is a measure to evaluate the accuracy of the model:
  3 $$\begin{aligned} e = L(t^\text{val}_{nc}, \textbf{X}^\text{val}) = -\sum ^{N^\text{val}}_{n=1}\sum ^{2}_{c=1} t^\text{val}_{nc} \ln p(c|\textbf{x}^\text{val}_n), \end{aligned}$$
  where $t^\text{val}_{nc}$ is the corresponding target value.
3. Shuffle the order of the *i*-th feature in the validation data (i.e., permutation) to create a new validation data matrix $\widetilde{\textbf{X}}^\text{val}$.
4. Input $\widetilde{\textbf{X}}^\text{val}$ into the trained model and compute the error $e_i = L(t^\text{val}_{nc}, \widetilde{\textbf{X}}^\text{val})$ based on the output.
5. Calculate the difference in the error before and after permuting *i*-th feature, $PI_i$, which is the evaluation value of permutation importance:
  4 $$\begin{aligned} PI_i = e_i - e. \end{aligned}$$
6. Repeat steps 3 to 5 for all features $i = 1,...,P$.

The above algorithm obtains the evaluation value of permutation importance $PI_i$ for each feature. Since the cross-entropy error is used as the score for evaluating the model’s accuracy, the larger $PI_i$ value indicates that the model’s accuracy deteriorates when the data order is randomized, suggesting that features with larger $PI_i$ are important.

### Relationship between evaluation indices and driving aptitudes

More than 300 indices were obtained from the 10 physical and cognitive function tests. Analyses of all these indices would require a great deal of time. Therefore, the indices that were considered to be related to driving aptitude were selected from all the indices under specialized physicians’ guidance. Indices that were highly related to driving aptitude were rated as $\circledcirc$, those that were related as $\bigcirc$, and those that were slightly related as $\triangle$. Table 2 shows the evaluation results for each test. The results were $\circledcirc$: 48 indices, $\bigcirc$: 17 indices, and $\triangle$: 7 indices. Moreover, the effective indices for driving aptitude evaluation were selected by machine learning using the proposed network using the three datasets created based on the evaluation results. Dataset 1 consisted of 48 indices with a rating of $\circledcirc$. Dataset 2 consisted of 65 indices with a rating of $\circledcirc$ and $\bigcirc$. Dataset 3 consisted of 72 indices with a rating of $\circledcirc$, $\bigcirc$, and $\triangle$. The data was standardized (mean = 0, standard deviation = 1) for each index. Table 3 shows the results of ROC analysis of the identification results by the proposed network. The AUC of dataset 1, dataset 2, and dataset 3 were 0.918, 0.946, and 0.862. Therefore, dataset 2 consisting of 65 indices of physical and cognitive functions listed in Table 4 were selected for analysis in this study (i.e., $P = 65$). The presence or absence of driving aptitude was used as the target value, and the values of 65 indices shown in Table 4 were used as input data. The experiments were run on a computer with an Intel Xeon X5-2620 (8 cores, 2.1$-$3.0 GHz) processor and 16 GB RAM.

**Table 2:** Evaluation results by physicians

	Inspection item										
	FIM	CAT	TMT	RBMT	MMSE	JPSS	BRS	BIT	HADS	AS	Total
$\circledcirc$	0	24	1	1	10	1	0	8	2	1	48
$\bigcirc$	0	10	0	0	0	0	7	0	0	0	17
$\triangle$	0	0	0	0	0	0	0	7	0	0	7

Physicians evaluate indices that are highly related to driving aptitude are rated as $\circledcirc$, those that are related as $\bigcirc$, and those that are slightly related as $\triangle$

**Table 3:** Results of ROC analysis based on datasets

Dataset	AUC	Sensitivity	Specificity	Threshold
Dataset 1	0.918	0.805	0.929	0.879
Dataset 2	0.946	0.878	0.929	0.830
Dataset 3	0.892	0.976	0.643	0.612

Sensitivity and specificity were calculated using the threshold values determined by the ROC analysis

The indices for driving aptitude evaluations were selected by machine learning using the proposed network, thereby judging the indices whose $w_i$ was non-zero at least once to be effective. In this study, 11-fold cross-validation was used for analysis. The ROC analysis was also conducted using the posterior probability of driving aptitude identified by LLGMN and the presence or absence of driving aptitude. The classification accuracy of driving aptitude by the selected indices was evaluated using AUC values. This study examines the validity of the indices selected based on the proposed method by comparing the results with those of permutation importance.

The evaluation value of permutation importance, $PI_i$, was calculated based on the 11-fold cross-validation using LLGMN on the dataset created with only the indices selected by the proposed network to verify whether the weight $w_i$ of the proposed network expresses the importance of the indices. Since the permutation importance calculates the evaluation value $PI_i$ for each fold of the cross-validation, 11 evaluation values can be obtained for each index *i*. This study calculates the average of $\{PI^{(1)}_i, PI^{(2)}_i, \ldots , PI^{(11)}_i\}$ obtained for each index in cross-validation as permutation importance for the final evaluation.

We also evaluated the prediction ability of the proposed method by comparing it with conventional methods. The LLGMN with dimensionality reduction by partial KLI [31, 34, 35] and Lasso regression were used as conventional methods. As an indicator of the importance of the selected indices, the reduction rate of the AUC value generated by deleting each index after dimensionality reduction was used in LLGMN with dimensionality reduction by partial KLI, and the magnitude of the standardized partial regression coefficient was used in Lasso regression.

**Table 4:** List of indices used in the analysis

Test	Indices	Test	Indices
CAT	Digit span forward	RBMT	RBMT profile
	Digit span backward	MMSE	MMSE: Orientation-time
	Tapping span forward		MMSE: Orientation-location
	Tapping span backward		MMSE: Attention and calculation
	Visual cancellation Kana time		MMSE: Recall
	Visual cancellation $\triangle$ time		MMSE: Naming
	Visual cancellation star time		MMSE: Repetition
	Visual cancellation figure time		MMSE: 3-stage command
	Visual cancellation Kana accuracy		MMSE: Writing
	Visual cancellation Kana hit rate		MMSE: Copying
	Visual cancellation $\triangle$ accuracy		MMSE total score
	Visual cancellation $\triangle$ hit rate	JPSS	JPSS total score
	Visual cancellation star accuracy	BRS	Upper limbs on discharge
	Visual cancellation star hit rate		Fingers on discharge
	Visual cancellation figure accuracy		Lower limbs on discharge
	Visual cancellation star figure rate		Dominant side Left
	SDMT number of wrong answers		Dominant side Right
	SDMT achievement rate		Affected side Left
	Memory updating 3 span accuracy		Affected side Right
	PASAT:2 seconds accuracy	BIT	Line crossing test
	Position Stroop accuracy		Letter cancellation test
	Position Stroop time		Star cancellation test
	CPTAX accuracy		Copying test
	CPTAX hit rate		Line bisection test
	CPTAX average reaction time		Drawing test
	CPTAX coefficient of validation		BIT conventional subtest
	CPTSRT accuracy		BIT behavioral subtest
	CPTSRT hit rate	HADS	Depression score
	CPTSRT average reaction time		Anxiety score
	CPTSRT coefficient of validation	AS	Apathy score
	CPTX accuracy	TMT	Part A time
	CPTX hit rate		
	CPTX average reaction time		
	CPTX coefficient of validation		

CAT: clinical assessment for attention; SDMT: Symbol digit modalities test; PASAT: Paced auditory serial addition test; CPTAX: Continuous performance test AX version; CPTSRT: Continuous performance test simple version; CPTX: Continuous performance test X version; TMT: Trail making test; RBMT: Rivermead behavioral memory test; MMSE: mini-mental state examination; JPSS: Japanese perceived stress scale; BRS: Brunnstorm stages of motor recovery; BIT: Behavioral inattention test; HADS: Hospital anxiety and depression scale; AS: Apathy score

The proposed network was trained using stochastic gradient descent (SGD) with a learning rate of 0.01, a batch size of 50, and a number of epochs of 10,000. The learning was terminated when the loss did not improve by more than $1\times 10^{-4}$ for more than 5 epochs. The regularized parameter $\lambda$ of the proposed network was optimized using the tree-structured Parzen estimator (TPE) [36]. The detailed settings of the TPE are described in Additional file 1.

### Statistical analysis

Fisher’s exact test was performed to confirm the relationship between the evaluation of driving aptitude by the indices selected by the proposed network and the evaluation by the instructor of the driving school at a significance level of 5%. The evaluation of driving aptitude by the indices selected by the proposed network was discriminated against using thresholds of the maximum sum of sensitivity and specificity attributed to the ROC analysis. Additionally, Yule’s correlation coefficient was calculated to confirm the strength of the relationship. The AUC values for the conventional methods and the proposed method were calculated and compared using the Holm-adjusted Delong test at a significance level of 5%.

## Results

In this study, we used 65 indices obtained from physical and cognitive function tests conducted on 55 participants with stroke (Table 4). These indices were input into a neural network model to evaluate driving aptitude. The proposed neural network model consists of a dimensionality reduction layer and a discriminative layer (Fig. 1). In the dimensionality reduction layer, there is a single weight parameter corresponding to each input index. The value of the weight parameter is determined according to the contribution of each corresponding input index to the prediction of driving aptitude. For a discriminative layer, we used the log-linearized Gaussian mixture network (LLGMN), which is a neural network based on discriminative Gaussian mixture models. During the model training, the weight parameters of the entire network were optimized based on the loss function with the $L_1$ norm on the weights of the dimensionality reduction layer. This learning method provides a sparse solution for the weight parameters of the dimensionality reduction layer, thereby effectively eliminating less important input variables in the learning process. Therefore, the proposed neural network model can find important evaluation indices as well as evaluate driving aptitude.

### Prediction accuracy of driving aptitude

**Fig. 2:**
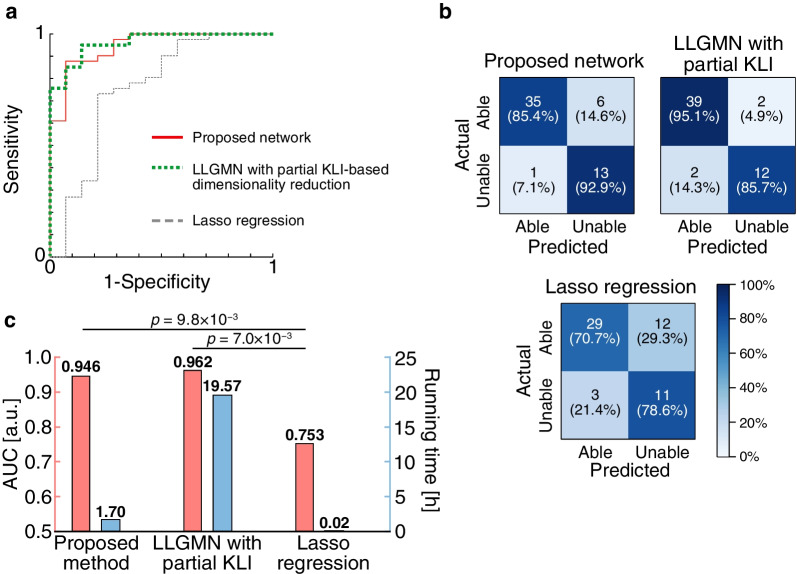
Results of driving aptitude evaluation. **a** ROC curve for each method. Red solid line, green dotted line, and gray dashed line represent the proposed network, LLGMN with partial KLI-based dimensionality reduction, and Lasso regression, respectively. **b** Confusion matrix for comparing the diagnostic results with the results analyzed by each method. **c** Comparison of predictive ability and time complexity of three methods. The statistical test results based on the Delong test with Holm adjustment are also shown (significance level: 0.01)

Dimensionality reduction and prediction of driving aptitude based on the proposed method were performed using 65 indices as input variables. The training and testing processes were conducted by 11-fold cross-validation. For comparison, we used two baselines: the LLGMN with dimensionality reduction by partial Kullback–Leibler information (KLI) and the least absolute shrinkage and selection operator (Lasso) regression. The partial KLI-based dimensionality reduction is a discrete variable selection method that reduces the input variables stepwise based on the class posterior probabilities output by LLGMN. The Lasso regression is a linear regression analysis method with $L_1$ regularization, which allows continuous variable selection. Each analysis’s duration was 1.7 h for the proposed network, 19.6 h for LLGMN with dimensionality reduction using partial KLI, and 0.016 h for Lasso regression, as shown in Fig. 2c.

Figure 2a and Table 5 show the receiver operating characteristic (ROC) analysis of each method. The area under the curve (AUC) of the proposed network and LLGMN with partial KLI-based reduction were both $> 0.94$, showing that these methods could discriminate driving aptitude with high accuracy. Significant differences between the proposed network and Lasso regression and between LLGMN with partial KLI-based reduction and Lasso regression were observed (proposed network vs. Lasso regression: $p=9.8 \times 10^{-3}$, LLGMN with dimensionality reduction using partial KLI vs. Lasso regression: $p=7.0 \times 10^{-3}$).

**Table 5:** Results of ROC analysis

Analysis method	AUC	Sensitivity	Specificity	Threshold
Proposed network	0.946	0.878	0.929	0.830
LLGMN with partial KLI-based reduction	0.962	0.951	0.857	0.730
Lasso regression	0.753	0.732	0.786	0.808

Sensitivity and specificity were calculated using the threshold values determined by the ROC analysis

Figure 2b shows the confusion matrices of the evaluation of driving aptitude by each method and the evaluation by the instructors of the driving school. There were significant relationships between the evaluations of each method and the instructors (proposed network: $p=2.3 \times 10^{-7}$, LLGMN with partial KLI-based reduction: $p=1.7 \times 10^{-8}$, and Lasso regression: $p=1.8 \times 10^{-3}$). Yule’s coefficient of association for each method was 0.974 (proposed network), 0.983 (LLGMN with partial KLI-based reduction), and 0.797 (Lasso regression), meaning that the neural network-based dimensionality reduction showed a high degree of relationships between actual and predicted driving aptitudes.

### Importance of indices for driving aptitude evaluation

The importance of each index can be evaluated by examining the values of the weight parameters, $w_i$, in the dimensionality reduction layer of the proposed network, since the $L_1$ regularization shrinks the weight parameters related to the indices with a low contribution to the driving aptitude. Therefore, the indices with larger values of the corresponding weight parameters can be interpreted as more important variables in evaluating driving aptitude.

**Fig. 3:**
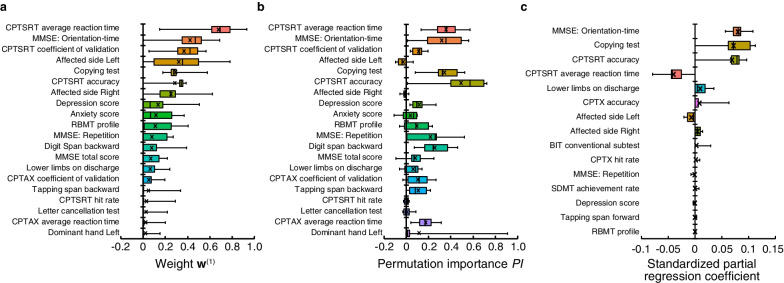
Results of importance evaluation of indices. **a** Optimized weights ($w_i$) of indices selected by the proposed network. **b** Results of permutation importance for indices selected by the proposed network. **c** Standard partial regression coefficients for Lasso regression. “$\times$” in the boxplot denotes the average value of each index

Resultantly, the original 65 input indices were reduced to 20 after dimensionality reduction using the proposed network, whose corresponding weight parameter values have never been reduced to zero due to the 11-fold cross-validation analysis. Figure 3a shows the boxplots of weight parameters, $w_i$, of the indices selected by the proposed network, sorted in descending order by their mean values. The index with the largest averaged $w_i$ was *CPTSRT average reaction time*. *MMSE: Orientation-time*, *CPTSRT coefficient of validation*, *Affected side Left*, and *Copying test* had the next largest averaged $w_i$, in that order. In addition, as shown in Table 1, four of these five indices were significantly different between the two groups with and without driving aptitude: *CPTSRT average reaction time* ($p=0.0499$), *MMSE: Orientation-time* ($p=0.0004$), *Affected side Left* ($p=0.0286$), and *Copying test* ($p=0.0058$).

We also conducted a similar analysis using permutation importance [33] as another way to evaluate the importance of indices using a learned model. Figure 3b shows the result of the calculated permutation importance, denoted as *PI*, using the 20 indices selected using the proposed network. The order of the indices was the same as in Fig. 3a. The index with the largest average *PI* is *CPTSRT accuracy*. *CPTSRT average reaction time*, *Copying test*, *MMSE: Orientation-time*, and *Digit span backward* were the next indices with the largest average *PI* in that order. These results showed that four out of the top five indices with the largest *PI* were consistent with the top six indices with the largest average $w_i$ of the proposed network. It is worth noting that the *CPTSRT accuracy* of the two groups of participants was also significantly different ($p=0.0168$) as shown in Table 1.

**Table 6:** Ranking list of the indices selected by LLGMN with partial KLI-based dimensionality reduction

Rank	Removed index	AUC	AUC reduction (%)
1	CPTSRT accuracy	0.810	15.8
2	CPTAX average reaction time	0.819	14.9
3	CPTSRT coefficient of validation	0.848	11.8
4	Digit span backward	0.854	11.2
5	Letter cancellation test	0.873	9.2
6	MMSE: Repetition	0.875	9.1
7	Anxiety score	0.885	8.0
8	CPTX coefficient of validation	0.887	7.8
9	MMSE: Orientation-time	0.889	7.6
10	BIT conventional subtest	0.901	6.3
11	Digit span forward	0.901	6.3
12	Dominant side Left	0.904	6.0
13	RBMT profile	0.904	6.0
14	TMT part A time	0.934	2.9
15	Visual cancellation Kana hit rate	0.937	2.5
16	Depression score	0.944	1.8
17	Dominant side Right	0.967	−0.5
18	Copying test	0.967	−0.5
19	CPTSRT average reaction time	0.977	−1.6

Indices are listed in order of increasing the AUC reduction

Table 6 shows the indices selected for dimensionality reduction by partial KLI and the reduction rate of AUC values after removing indices in descending order. Nineteen indices were obtained after dimensionality reduction by partial KLI. The maximum reduction in the AUC was 15.8% of *CPTSRT accuracy*. Additionally, the decrease in the AUC for *CPTAX average reaction time* was 14.8%, for *CPTSRT coefficient of validation* was 11.8%, for *Digit span backward* was 11.2%, and for *Letter cancellation test* was 9.2%. Figure 3c shows the mean of the standardized partial regression coefficients of Lasso regression in descending order of absolute value. The standardized partial regression coefficients were largest for *MMSE: Orientation-time*; the next largest coefficients were for *Copying test*, *CPTSRT accuracy*, *CPTSRT average reaction time*, and *Lower limbs on discharge*. The top five most important indices for each analysis method were *MMSE: Orientation-time* of MMSE, 2 sub-tests (*CPTSRT average reaction time* and *CPTSRT accuracy*) of continuous performance test (CPT) of clinical assessment for attention (CAT), and 2 sub-tests (*Copying test* and *Letter cancellation test*) of behavioral inattention test (BIT) (Fig. 3 and Table 6). These results indicated that the same indices were selected by all analysis methods.

## Discussion

In this paper, we present a neural network model with a dimensionality reduction layer and analyzed the physical and cognitive function test results of post-stroke individuals associated with driving aptitude. In the proposed network, $L_1$ norm-based sparse regularization is applied to the weight parameters of the dimensionality reduction layer, and the entire network is trained end-to-end for continuous variable selection of input indices. Using the various indices of each participant as input data and the corresponding driving aptitude as output labels, the proposed approach enables the automatic extraction of important indices for evaluating the driving aptitude of post-stroke individuals out of a huge number of medical indices.

The AUC values of the indices selected by the proposed network and the indices selected by dimensionality reduction with partial KLI showed high accuracy in discriminating driving aptitude (Table 5), whereas that of Lasso regression showed lower accuracy. Fisher’s exact test showed a significant relationship for each method, but Yule’s coefficient of relationship for the proposed network and LLGMN with dimensionality reduction using partial KL information were higher than that for Lasso regression, reaching $>0.97$. This suggests that the nonlinear analysis methods, the proposed network and LLGMN with dimensionality reduction using partial KL information, are more suitable for analyzing the relationship between physical and cognitive function tests and driving aptitude compared with a linear analysis method, Lasso regression. In addition, the analysis time of the proposed network is much shorter than that of the partial KLI-based method (Fig. 2c). This is because the proposed network can select indices continuously by end-to-end training, while the partial KLI-based method selects indices stepwise by repeating the training for the number of indices.

Four of the top five indices with the largest *PI* coincided with the top six indices with the largest average weight $w_i$ of the proposed network (Fig. 3a and b), all of which are also significantly between the two groups of participants with and without driving aptitude. This suggests that the average weights $w_i$ of the proposed network reflect the importance of the indices in terms of their contribution to discrimination. In contrast, some indices, such as *Affected side Left*, have a large average of weight $w_i$ without a large *PI*. The distribution of $w_i$ was confirmed to be wide by focusing on the weight $w_i$ of *Affected side Left*. This suggests that the *PI* became small even for indices with a large average of $w_i$ because the weights for each fold in the cross-validation differed greatly. However, this *Affected side Left* suggests the importance of right hemisphere damage in determining driving aptitude. In our previous studies, the right hemisphere has been shown to have cognitive functions related to car driving, directed attention, and sustained attention. In addition, our previous research has shown that the left and right hemispheres of the brain can coordinate to regulate the speed and accuracy of thought and action processing [27]. Therefore, both left and right affected sides were included in the remaining indices in the evaluation.

For each analysis method, *MMSE: Orientation-time*, *CPTSRT average reaction time*, *CPTSRT accuracy* and other indices obtained by the CPT, and the *Copying test* and *Letter cancellation test*, which are indices obtained by the BIT, were selected (Fig. 3, Table 6). This consistent result suggests that the indices selected by the proposed method are appropriate since the proposed method can select indices similar to those of the conventional methods. The relationship between these indices and driving aptitude is discussed below. *MMSE: Orientation-time* is an index to evaluate the orientation of time. This index may be selected as an index of declining cognitive function because conventional research has reported that declining orientation of time is an early stage of declining cognitive function required for driving [37]. The CPT, a sub-test of the CAT, evaluates sustained attention. Sustained attention is considered an index related to driving aptitude. It has been reported that slow cognitive processing speed related to sustained attention [38] is associated with traffic accidents [39]. *Copying test* and *Letter cancellation test* which are sub-tests of BIT are obtained. BIT is used for the purpose of measuring unilateral spatial neglect which has been known to interfere with driving a car [40]. For these reasons, the indices selected for each analysis method are related to driving aptitude and are considered important indices for evaluating driving aptitude. Typically, it takes a total of about 5 h to complete all 10 physical and cognitive function tests, which can last one week or more. However, if the number of indices can be reduced based on the results of this study, effective evaluation of driving aptitude can be achieved by completing the physical and cognitive function test in one day. In conclusion, the proposed method provides compelling evidence for the effective evaluation of driving aptitude in individuals with stroke by specifying highly relevant tests of physical/cognitive functions and reducing the number of tasks required in the screening test, potentially raising the possibility of their resumption of car driving as well as reducing the risk of accidents.

### Limitations and future work

In this paper, we evaluated driving aptitude in 55 participants with stroke using the developed machine learning model. Whereas current work has narrowed down evaluation indices that would have taken a week to obtain to the extent of a single day, it remains challenging to justify a full day of driving assessment for participants with stroke. In addition, the specificity of all three investigated classification methods may be questioned in the clinical setting by the 4.9$-$29.3% of false positives.

On the one hand, the dataset of the same participants was used for the cross-validation to select indices and for the cross-validation to evaluate the driving aptitude with the selected indices due to the small number of participants enrolled. Therefore, the evaluation of driving aptitude in this study is not a rigorous evaluation of completely unknown data. In the future, priority should be given to increasing the number of participants and confirming the evaluation accuracy of unknown data.

On the other hand, previous studies have reported an association between motor-cognitive functions and the location of brain damage [27]. Therefore, we intend to include brain imaging analysis in the future to identify brain regions associated with driving aptitude, which may contribute to higher specificity while maintaining the evaluation duration, thus improving the clinical feasibility. Furthermore, although the *w* of the dominant side factor before stroke is low, it is still possible that driving ability was related to whether the affected side was on the dominant side. In the future, we would like to clarify this relationship by analyzing lesion sites.

## Conclusion

This paper demonstrated that the sparse neural network, LLGMN with $L_1$ dimensionality reduction layer, can analyze physical and cognitive function test results associated with driving aptitude more efficiently than the conventional method and identifies the presence or absence of driving aptitude with high accuracy. In addition, the indices obtained from the MMSE and the CPT, which is a sub-test of the CAT and the BIT, were important in the evaluation of driving aptitude. This indicates the possibility of evaluating driving aptitude with fewer tests than those that are currently required.

### Supplementary Information

**Additional file 1:** Optimization method for regularization parameters.

## Data Availability

The datasets supporting the conclusions of this article are available on request and if no ethical reasons prohibit sharing.

## Abbreviations

ADLs: Activities of daily living
AS: Apathy score
AUC: Area under the curve
BIT: Behavioral inattention test
BRS: Brunnstorm stages of motor recovery
CAT: Clinical assessment for attention
CPT: Continuous performance test
CPTAX: Continuous performance test AX version
CPTSRT: Continuous performance test simple version
CPTX: Continuous performance test X version
HADS: Hospital anxiety and depression scale
JPSS: Japanese perceived stress scale
KLI: Kullback–Leibler information
Lasso: Least absolute shrinkage and selection operator
LLGMN: Log-linearized Gaussian mixture network
MMSE: Mini-mental state examination
PASAT: Paced auditory serial addition test
RBMT: Rivermead behavioral memory test
ROC: Receiver operating characteristic
SDMT: Symbol digit modalities test
SGD: Stochastic gradient descent
TMT: Trail-making test
TPE: Tree-structured Parzen estimator
