# Supplementary material for: Motor-cognitive functions required for driving in post-stroke individuals identified via machine-learning analysis
Source: J Neuroeng Rehabil. 2023 Oct 18;20:139. doi: 10.1186/s12984-023-01263-z (PMC10583407; doi:10.1186/s12984-023-01263-z)
Supplement: Supplementary file 1 — Additional file 1: Optimization method for regularization parameters. [file 12984_2023_1263_MOESM1_ESM.pdf]

Supplementary Information for

Motor-Cognitive Functions Required for Driving in Post-Stroke Individuals Identified via Machine-Learning Analysis

Genta Tabuchi, Akira Furui, Seiji Hama, Akiko Yanagawa, Koji Shimonaga, Ziqiang Xu, Zu Soh, Harutoyo Hirano, Toshio Tsuji

*Corresponding author(s). E-mail(s): shama@hiroshima-u.ac.jp; tsuji-c@bsys.hiroshima-u.ac.jp;

Supplementary Note

Optimization method for regularization parameters

Statistical distribution estimation for a high-dimensional input space ($\mathbf{X}(n) \in \mathbb{R}^P$) is prone to local solutions. One method to prevent this is L_1 regularization. The L_1 regularization learns to minimize the sum of the loss function values and the weights. Thus, the L_1 regularization prevents the weights from becoming too large. However, optimization is necessary because the regularization strength varies depending on the size of the regularization parameter λ. In this study, the tree-structured Parzen estimator (TPE) [1] was used as an optimization method. The TPE performs optimization by the following algorithm.

1. Set the number of random search C and the number of search by TPE M.
2. Perform a random search of the search point λ in C times and calculate the corresponding loss y.
3. Set the threshold y^* such that the number of search points λ with $y \leq y^*$ is γ % of the total, and divide the search points λ into groups L ($y \leq y^*$) and G ($y > y^*$).
4. For the magnitude of y, they are arranged in ascending order for group L and descending order for group G. Estimate the probability density function for each using the following equation.

$$p(\lambda|y) = \begin{cases} l(\lambda) = \frac{1}{m}\sum_{j=1}^{m} h_j^l K\left(\lambda|\lambda_j^l, \sigma_j^l\right) & (y \leq y^*), \\ g(\lambda) = \frac{1}{m}\sum_{j=1}^{m} h_j^g K\left(\lambda|\lambda_j^g, \sigma_j^g\right) & (y > y^*), \end{cases} \tag{1}$$

   where m is the number of search points, $K(\lambda|\lambda_j, \sigma_j)$ is the kernel density function, σ_j^l and σ_j^g are the bandwidths, and h_j^l and h_j^g are the coupling coefficients for the kernel density function.

5. Take several candidate points at random and calculate the expected amount of improvement in loss using the following equation:

$$EI(x) = \frac{l(\lambda)}{g(\lambda)}. \tag{2}$$

6. The candidate point with the highest EI is set as the next search point, and calculate y.
7. Repeat steps 2 to 5 in M times.

The above algorithm was used to optimize the regularization parameters. For the final regularization parameter λ, we applied the value that minimizes y.

In this study, the kernel density function K is Gaussian function, parameters are $C = 20$, $M = 30$, $\gamma = 25$, search range is $0.001 < \lambda < 1$, loss is $y = 1 - AUC$, respectively. The coupling coefficients h_j^d and σ_j^d ($d \in \{l, g\}$) were defined as following equations [2]:

$$h_j^l = 1, h_j^g = \begin{cases} \frac{e_{\max}^g - j}{e_{\max}^g - 26} & (j \geq 26), \\ 1 & (otherwise), \end{cases} \tag{3}$$

$$\sigma_j^d = \begin{cases} \lambda_1^d - \lambda_{\min}^d & (j = 1), \\ \max(\lambda_{j+1}^d - \lambda_j^d, \lambda_j^d - \lambda_{j-1}^d) & (1 < j < e_{\max}^d), \\ \lambda_{\max}^d - \lambda_{e_{\max}^d}^d & (j = e_{\max}^d), \end{cases} \quad (4)$$

where $\lambda_{\min}$, $\lambda_{\max}$ and $e_{\max}$ are the minimum, maximum, and number of elements in each group, respectively.

References

[1] Bergstra, J., Bardenet, R., Bengio, Y., Kégl, B.: Algorithms for hyper-parameter optimization. Advances in Neural Information Processing Systems **24**, 2546–2554 (2011)

[2] Bergstra, J.: Hyperopt. (Accessed on January 22, 2021). https://github.com/jaberg/hyperopt
